# Lignocelluloytic activities and composition of bacterial community in the camel rumen

**DOI:** 10.3934/microbiol.2021022

**Published:** 2021-09-24

**Authors:** Alaa Emara Rabee, Robert Forster, Ebrahim A Sabra

**Affiliations:** 1 Animal and Poultry Nutrition Department, Desert Research Center, Cairo, Egypt; 2 Lethbridge Research and Development Centre, Agriculture and Agrifood Canada, Lethbridge, AB, Canada; 3 Animal Biotechnology Department, Genetic Engineering and Biotechnology Research Institute, University of Sadat City, Sadat City, Egypt

**Keywords:** Camel rumen, bacteria, archaea, enzymes, cellulase, xylanase

## Abstract

The camel is well-adapted to utilize the poor-quality forages in the harsh desert conditions as the camel rumen sustains fibrolytic microorganisms, mainly bacteria that are capable of breaking down the lignocellulosic biomass efficiently. Exploring the composition of the bacterial community in the rumen of the camel and quantifying their cellulolytic and xylanolytic activities could lead to understanding and improving fiber fermentation and discovering novel sources of cellulases and xylanases. In this study, Illumina MiSeq sequencing of the V4 region on 16S rRNA was applied to identify the bacterial and archaeal communities in the rumen of three camels fed wheat straw and broom corn. Furthermore, rumen samples were inoculated into bacterial media enriched with xylan and different cellulose sources, including filter paper (FP), wheat straw (WS), and alfalfa hay (AH) to assess the ability of rumen bacteria to produce endo-cellulase and endo-xylanase at different fermentation intervals. The results revealed that the phylum Bacteroidetes dominated the bacterial community and *Candidatus Methanomethylophilus* dominated the archaeal community. Also, most of the bacterial community has fibrolytic potential and the dominant bacterial genera were *Prevotella*, *RC9_gut_group*, *Butyrivibrio*, *Ruminococcus*, *Fibrobacteres*, and *Treponema*. The highest xylanase production (884.8 mU/mL) was observed at 7 days. The highest cellulase production (1049.5 mU/mL) was observed when rumen samples were incubated with Alfalfa hay for 7 days.

## 1. Introduction

The camel (*Camelus dromedaries*) produces milk and meat under desert conditions more than other ruminants [1]. This unique animal is well adapted to hot desert conditions by its unique feeding behavior and the functional structure of its digestive tract [2]. The digestion in the rumen depends on the microbial fermentation that takes place in the rumen, the first compartment in the camel stomach [3]. The retention time of feed particles in the camel rumen is longer than other ruminants, which prolongs the exposure of lignocellulosic biomass to the fibrolytic microorganisms that helps the efficient digestion [4]–[6]. Moreover, the high-digestion efficiency of the camel rumen could be attributed to the structure of the microbial community in the rumen, where the lignocellulolytic bacteria dominate the microbial community in the rumen of the camel [7]. This finding is supported by a metagenomics analysis in the camel rumen microbiome, which revealed that the camel rumen microbiome contains higher proportions of glycoside hydrolases compared with other gastrointestinal metagenomes from other herbivores [8],[9].

Therefore, the camel rumen microbiota could be a rich source of cellulase and xylanase enzymes that could be used in a wide range of biotechnological and industrial applications [10]. Bacteria dominate the microbial community in the rumen and make the greatest contribution to rumen fermentation [11]. Also, archaea remove the hydrogen (H_2_) in the rumen by using it to reduce carbon dioxide (CO_2_) to methane (CH_4_) through methanogenesis [7]. The production of methane increases greenhouse gases emissions [12] and represents a loss in dietary gross energy intake [13]. Therefore, investigation of these microbial communities is the key to understanding their roles and maximize ruminal fermentation and fiber digestion [14]. The chemical composition of the animal diet is the main determiner of the structure and abundance of rumen microbiota [1],[3]. For instance, poor-quality feeds that are rich in lignocellulose, including wheat straw stimulate the fibrolytic bacteria and starchy feeds stimulate amylolytic bacteria [7].

Many rumen bacterial isolates are involved in the production of cellulolytic enzymes commercially such as *Rumminococcus* [15], *Bacillus* [16],[17], *Clostridium* [18]. Therefore, the camel rumen has received great interest for mining for enzymes with biotechnological and industrial applications [9],[10],[19],[20]. Cellulases and xylanases have a key role in the bioconversion of lignocellulosic biomass to animal feed or fermentable sugars for bioethanol production [21]. Lignocellulolytic enzymes are widely used in feed additives to improve the animal digestibility and gut health [22]. Furthermore, these enzymes have many industrial and biotechnological applications such as in textiles and detergent industry, and food and pharmaceutical applications [17],[23]. Therefore, the demand for cheap, high-active, and stable enzymes are growing rapidly [10],[21].

There is a need to understand the ability of camels to utilize the poor-quality forages with a high content of lignocellulose [5],[6] and to discover novel sources of lignocellulolytic enzymes [24]. Therefore, this study aims to explore the composition of the bacterial community in the rumen of camels fed wheat straw and broom corn and to assess the ability of the camel rumen anaerobic bacteria to produce cellulase and xylanase enzymes using different cellulose sources.

## 2. Materials and methods

### 2.1. Animals and sampling

Camels in this study (n = 3) were reared in a commercial private farm in Giza, Egypt. They were housed in shaded pens and fed wheat straw and broom corn and offered free drinking water. Then the camels were slaughtered in the Giza slaughtering house, Giza, Egypt. The chemical compositions of wheat straw and broom corn are presented in supplementary table S1. The rumen samples were obtained after slaughtering and were strained via cheesecloth. Apart of liquid samples were cryopreserved using glycerol according to the protocol of Phillips and Gordon. (1988) [25] for cultivation purposes and 5 mL from every liquid sample were frozen using liquid nitrogen and stored at −80 °C for further processing.

### 2.2. RNA isolation, PCR amplification, and amplicon sequencing

Total RNA was extracted and reverse-transcribed into cDNA using the protocol of Wang et al. (2017) [26]. The 16S rRNA gene was amplified using primer set 338F and 806R for V4 region [27]. The PCR amplifications were performed by PTC-220 DNA Engine Dyad Peltier Thermal Cycler, Roche Molecular system. The PCR reaction contained mix of 4 µL template cDNA, 12.5 µL KAPA2G Robust Hot Start ready mix PCR kit (KAPA BIO), 1.25 µL of forward primer, 1.25 µL of reverse primer, and 6 µL molecular biology water. The cycling conditions were, 1 cycle at 95 °C for 3 min and 30 cycles at 94 °C for 20 s, 65 °C for 20 s and 72 °C for 50 s followed by 72 °C for 3 min. The PCR products were gel-purified using QIAquick Gel Purification Kit (Qiagen) and DNA concentration was measured using Quant-iTPico Green dsDNA Assay Kit (Invitrogen). Then, the libraries were finally quantified by 7900HT Fast Real-Time PCR System (Life Technologies Corporation) using NEBNext Library Quant Kit protocol. The libraries' amplicons were then sequenced in the Illumina MiSeq system using MiSeq Reagent Kit v2.

### 2.3. Data analysis

The analysis of libraries was performed using QIIME Version 1.9.0 [28]. The quality of generated 2 × 250 paired-end sequence reads was checked using Fast QC version 0.11.4 [29]. The adaptors, barcodes, and low quality reads were removed using Trimmomatic program version 0.35 [30]. Pear version 0.9.6 [31] was used to merge read1 and read2 in a single dataset. A de novo picking of Operational Taxonomic Units (OTUs) was performed using SILVA databases as references. Alpha diversity indices, Chao1, Shannon, inverse Simpson's, and the number of OTUs were calculated using QIIME. All Sequences have been deposited in SRA under study code SRP105269 with the accession numbers SRX2765886, SRX2765885, and SRX2765884.

### 2.4. Cultivation condition

The growth medium that was used in this experiment was the modification of Medium 10 [32]. The composition of the growth medium was as follow (per 1000 ml distilled water): 2 g trypticase, 0.5 g yeast extract, 37 mL solution of K_2_HPO_4_•3H_2_O (0.6 g in 100 mL distilled H_2_O), 37 mL salt solution [0.16 g CaCl_2_•2H_2_O, 0.6 g KH_2_PO_4_, 1.2 g NaCl, 0.6 g (NH_4_)_2_SO_4_, 0.25 g MgSO_4_•7H_2_O in 100 mL distilled H_2_O], 1 mL Hemin solution (1 g L-1), 1mL Resazurin solution (1 g L-1), 50 mL solution of Na_2_CO_3_ (8 g in 100 distilled H_2_O), 1 g L-cysteine HCl, 200 mL clarified rumen fluid, 1 mL vitamin mix and 1mL trace mineral solution that were described by McSweeney et al. (2005) [33]. Also, clarified rumen fluid and anaerobic medium were prepared according to the protocol of McSweeney et al. (2005) [33]. To measure the xylanolytic activities of rumen bacteria, the growth media were enriched with birchwood xylan (100 mg/bottle) (X). To determine the cellulolytic activities, the growth media were enriched with one of three fiber sources, filter paper (FP) (2 discs/bottle), wheat straw (WS) (100 mg/bottle), and alfalfa hay (AH) (100 mg/bottle). The pH was adjusted at 6.8 and the media were prepared under anaerobic condition. Anaerobic medium (50 mL) was tubed into 120 mL-Serum bottles under steam of CO_2_; then the bottles were sealed and autoclaved at 121 °C for 15 min. Eight bottles were prepared for every sample for four media (X, FP, WS, and AH) (3 animals, 2 replicates and 4 media; 8 bottles per animal). Preserved rumen samples were thawed by warm water, and then 0.3 mL was inoculated to the growth media. The inoculated bottles were incubated at 39 °C and the bacterial growth was checked by the microscopic examination and the degradation of filter paper. Aliquots for enzyme measurement at three time intervals, 24 h, 48 h, and 7days were collected.

### 2.5. Cellulase and xylanase enzyme assay

Samples of growing cultures were collected at different time intervals as shown previously. The collected samples were centrifuged at 13000 xg, 10 min, 4 °C and the supernatant served as the enzyme source. Cellulase and xylanase activities (mU/mL) were measured using EnzChek Cellulase substrate kit (Invitrogen, UK) that determines endo-1,4-β-glucanase and EnzChek Ultra Xylanase Assay Kit (Invitrogen, UK) that determines endo-1,4-β-xylanase according to the manufacturer recommendations and a blank of media without inoculation was used.

### 2.6. Statistical analysis

The statistical analyses were performed using the IBM SPSS20 version 20 [34], and the Tukey test was carried out to determine the significant differences at p < 0.05. The difference in xylanase production at different incubation times was performed using Repeated Measures ANOVA and the differences in cellulase production using different cellulose sources at different incubation times were performed using Mixed ANOVA.

## 3. Results

### 3.1. Sequencing information and diversity indices

The sequencing of variable region 4 (V4) of 16S rRNA in three rumen samples resulted in 35310 high-quality sequence reads. The total number of sequence reads was 13450 in animal A, 11770 in animal B and 10090 in animal C. A total of 8329 OTUs were observed in the three samples with a total of 3258 OTUs were detected in animal A, 2455 in animal B, and 2616 in animal C. Alpha diversity analysis of the microbial community was performed using different indices, including Chao1, Shannon, Inverse Simpson and Phylogenetic Diversity (PD) Whole tree (Table 1). The sequence reads in the current study were identified as bacteria (94.58%), archaea (1.07%), and 4.35 % of sequence reads were not assigned to any specific microbial group.

**Table 1.** Alpha-diversity indices of microbial community in the rumen of camels.

	Animal A	Animal B	Animal C	Overall mean
PD_whole_tree	166.858	151.181	151.966	156.6
Chao1	11885.3	8959.611	10111.83	10318.9
Observed OTUs	3258.4	2455.1	2616.2	2776.5
Shannon	8.826	7.959	8.843	8.54
Simpson	0.986	0.976	0.986	0.98

**Figure 1.**
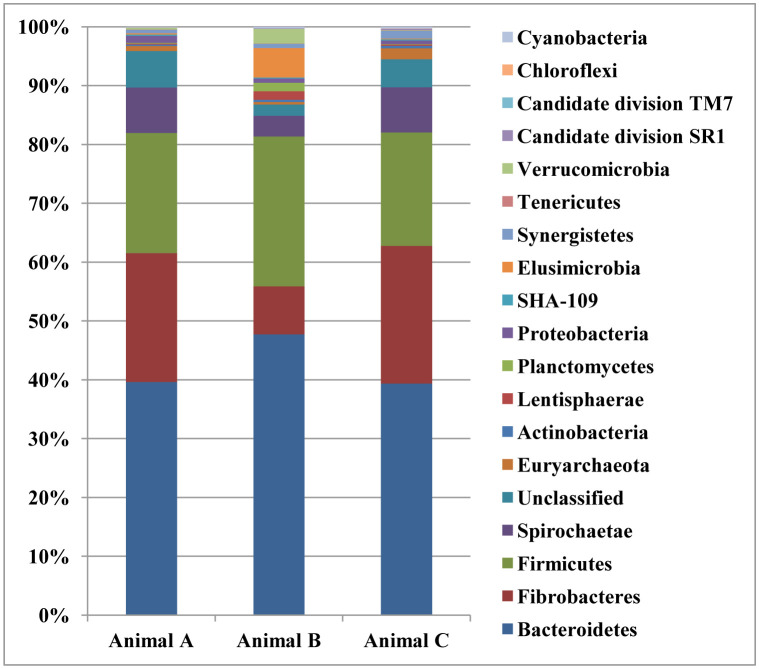
Stacked bar chart shows the relative abundance of bacterial phyla in animal A, B, and C.

**Figure 2.**
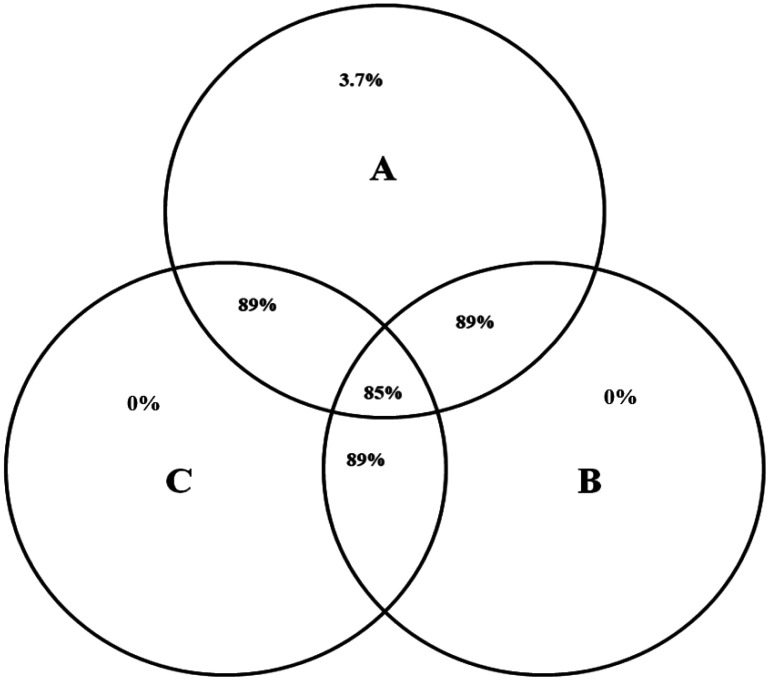
Venn diagram shows the number of bacterial genera shared between rumen samples of camel A, B and C. Each circle represents an animal and the overlapping areas represent the common bacterial genera.

In the current study, seventeen bacterial and one archaeal phyla were observed in the rumen of camels under investigation. Phylum Bacteriodetes and Firmicutes represented about 75% of bacteria community. Other bacterial phyla that represented more than 0.8% of bacterial community were Fibrobacteres, Spirochaetae, and Elusimicrobia, Proteobacteria , Synergistes and Verrucomicrobia. Additionally, other phyla that were detected to be less than 0.8% were Actinobacteria, Candidate division SR1, Candidate division TM7, Cyanobacteria, Chloroflexi, Lentisphaerae, Planctomycetes, SHA-109, and Tenericutes (Figure 1; Supplementary Table S2). The bacterial community in the rumen of camels under investigation was assigned into 54 bacterial genera (Supplementary table S3). Venn diagram showed that 48 bacteria genera (85%) were shared between the three animals (Figure 2).

When the microbial community was inspected for family and genus level, the results revealed that phylum Bacteriodetes was dominated by family Prevotellaceae (27%), and genus *Prevotella*. Also, uncultured Bacteriodetes such as *RC9 gut group*, S24-7, BS11 gut group represented a high proportion (8.46%) of phylum Bacteriodetes. Phylum Firmicutes was dominated by family Lachnospiraceae (7.9%), which was dominated by genus *Butyrivibrio* and family Ruminococcaceae (10.32%), which was dominated by genus *Ruminococcus* (Supplementary table S3). Phylum Actinobacteria was dominated by the genus *Atopobium* and phylum Proteobacteria was dominated by *Desulfovibrio*.

The archaeal community in the rumen of camels under investigation was represented in four genera *Candidatus Methanomethylophilus* (0.81%), *Methanobrevibacter* (0.2%), *Methanosphaera* (0.04%), *Methanomicrobium* (0.01%) (Supplementary Table S3).

### 3.2. Lignocellulolytic enzymes production

This study investigated the ability of the bacterial community in the rumen of camels to produce cellulase and xylanase enzymes using rumen samples of camels fed wheat straw.

### 3.3. Xylanase production

**Figure 3.**
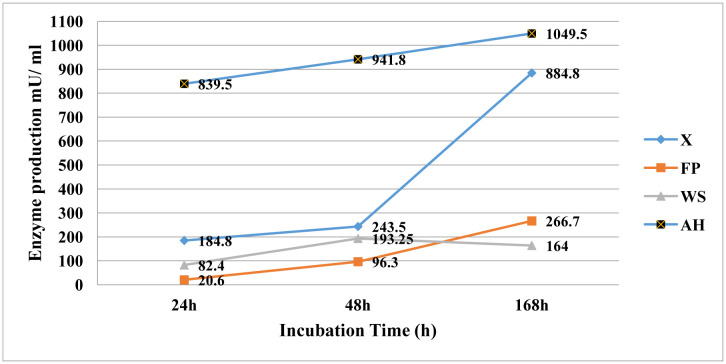
Effect of incubation time on xylanase and cellulase production by rumen bacteria of dromedary camel using birchwood xylan (x) and different cellulose sources, filter paper (FP), wheat straw (WS), and alfalfa hay (AH). Data are shown as means of three samples and asterisk shows the significant different differences at p < 0.05.

The bacterial xylanase (endo-1,4-β-xylanase ) production was measured at different incubation times by inoculating camel rumen samples into anaerobic bacterial medium containing birchwood xylan for 24 h, 48 h, and 7 days at 38 °C and pH = 6.8. The results revealed that xylanase production was increased from 24 h to 7 days. The overall mean production was 184.8 ± 101.3 mU/mL, (mean ± SD) at 24 h, 243.5 ± 68 at 48 h, and 884.8 ± 111.3 at 7 days. The difference in xylanase production at different fermentation times was significant (p < 0.01) (Figure 3).

### 3.4. Cellulase production at different incubation time and using different cellulose sources

Bacterial cellulase (endo-1,4-β-glucanase) production was quantified by inoculating the camel rumen samples into media containing one of three different cellulose sources, FP, WS, and AH at different incubation times 24 h, 48 h, and 7 days at 38 °C and Ph = 6.8. The results showed that cellulose sources impacted the cellulase production; also prolonging the incubation time increased the cellulase production and the highest activity was obtained at 7 days except for the WS media, where the production declined after 48 h (Figure 3; Supplementary Table S4). Regarding the substrate type, the maximum production was obtained with AH media at 7 days and the lowest production was observed with FP media at 24 h. The difference between incubation times and substrate type was significant (p < 0.01) and the interaction between substrate and incubation time was not significant.

## 4. Discussion

Lignocellulosic biomass could be hydrolyzed into fermentable sugars in the animal rumen by different types of cellulases and xylanases, which work synergistically to break down the cellulose and xylan in the plant cell wall [16]. The microbial communities in the rumen of dromedary camels are predominated by fibrolytic bacteria that make the greatest contribution to the fermentation of poor-quality plant biomass [3],[7]. Therefore, understanding the composition of rumen bacteria in the camels and their ability to produce cellulolytic and xylanolytic enzymes could open the door to maximizing animal production by improving lignocellulose degradation as well as discovering novel sources of enzymes with a wide range of applications [21]. Previous studies that were conducted on microbial community in the rumen of dromedary camels focused on the composition of the bacterial community using 16S rRNA/rDNA sequencing [1],[3],[7]. However, there is a need to determine the ability of the bacterial community to produce lignocellulolytic enzymes. Therefore, the current study explained the composition of bacteria and archaea in the rumen of three camels fed wheat straw and broom corn using cDNA-amplicon sequencing by Illumine MiSeq platform. Furthermore, the ability of rumen bacteria to produce xylanase and cellulase enzymes was determined. Alpha diversity indices were similar to values were observed in cattle [11],[35] and higher than values of Surti Buffalo [36]. Most of the bacterial reads (65.29%) were assigned to the Firmicutes and Bacteroidetes phyla (Figure 1), which is in agreement with previous studies on the camel rumen [1],[7], cattle [35], and Surti Buffalo [36]. Also, phylum Bacteroidetes dominated the bacterial community in this study, which is in agreement with the results of the camel rumen [3].

Bacteroidetes degrade the protein and polysaccharides such as cellulose, pectin, and xylan [37]. Uncultured members of Bacteroidetes are specialized in lignocellulose degradation [38]. Members of Bacteroidetes were dominated by *Prevotella* and *RC9_gut_group*; these results are similar to previous findings on bovines and camels [3],[39]. The *Prevotella* degrade hemicelluloses, pectin, starch, and protein and produce propionate in the rumen [40], which is used as an energy source by the host animal and declines the methanogenesis in the rumen [41],[42]. Thus, Bacteroidetes might play a key role in the utilization of poor-quality feeds in the rumen. Phylum Firmicutes was dominated by Ruminococcaceae and Lachnospiraceae families that were found to be active in fiber digestion in the rumen [37],[41]. Also, this phylum was dominated by cellulolytic bacterial genera, *Butyrivibrio* and *Ruminococcus* [3],[43].

The *Fibrobacteres* were observed in a higher proportion in the camel rumen. The percentage of this phylum in the current study was 18.1%, while it was 4.5–29% in Mehshana buffalo [37], 4.2–14.1% in wild ruminant [44], 3.09% in the camel in Iran [3]. *Fibrobacteres* have been shown in previous studies to be the principal cellulolytic active bacteria in the rumen [41],[45]. Genus *Treponema*, the dominant genus in phylum Spirochaetes, has the ability of cellulose degradation [46],[47]. Genus *Elusimicrobium* dominated the phylum Elusimicrobia; this genus was observed in the gut of cellulose-degrading termite [48]. Therefore, this phylum has a potential role in fiber degradation in the rumen [7]. Actinobacteria phylum has acetogenic activities and was found also in the rumen of moose [49]. Lentisphaerae phylum was dominated by *Victivallis* that ferment cellobiose [50].

The dominant bacterial families in this study were family Prevotellaceae (27%), uncultured Bacteriodetes (*RC9 gut group*, S24-7, BS11 gut group), and family Lachnospiraceae. All these groups have fibrolytic or potential fibrolytic activities [3],[7],[51],[52], which, indicates that most of the bacterial community (about 80%) in the rumen of the camels under investigation has a role in fiber degradation. This could explain the ability of camels to survive in desert harsh conditions with low-quality forages. Also, this result highlights the camel rumen as a good source of fibrolytic enzymes and productive bacteria [9],[10],[53].

The composition of the microbial community in the rumen is mainly influenced by the type of animal diet [54], and lignocellulolytic diets stimulate the fibrolytic microbes [7],[55]. The camels in this study were fed wheat straw, which is considered poor-quality forage as it has low nutritive value regarding crude protein, and soluble carbohydrate and it has high lignocellulose content [56], which might support the high proportion of fibrolytic bacteria.

This study explained the possibility of using the anaerobic bacterial community of the camel rumen in producing cellulase and xylanase by inoculating the camel rumen contents into anaerobic bacterial media enriched with xylan and different fiber sources, including filter paper, wheat straw, and alfalfa hay.

The maximum xylanase production (884.8 mU/mL) was observed at 7 days (Figure 3), this finding had a similar trend to results on different xylanolytic gut bacteria [57]. On the other hand, the anaerobic bacterial community in this study produced more xylanase than the aerobic fungi [58] and anaerobic rumen fungi of the camel gut [21]. Cellulase production by anaerobic bacteria in this study varied greatly between incubation periods and cellulose sources, which is in agreement with previous studies [15],[59]. In this study, we used three fiber sources, filter paper (FP), wheat straw (WS), and alfalfa hay (AH). The highest cellulase production (1049.5 mU/mL) was observed by anaerobic bacteria incubated in AH media at 7 days, similar results were obtained by the cellulolytic bacteria isolated from goat and swine [16],[57],[60], and cow manure [17]. Cellulase production by anaerobic bacterial community in the current study was higher than the production of *Bacillus* isolated from cow dung [17], cellulolytic bacteria isolated from goat and swine [57], and aerobic and anaerobic fungi [24],[58]. The decrease in production in WS after 48 hrs could be attributed to the depletion of nutritional ingredients in the medium [60].

In addition, this study identified the archaeal community in the camel rumen. Understanding the archaeal community in camel rumen is important as the methanogenic archaea are the main producers of methane in the rumen by converting the H_2_ and CO_2_ produced in the rumen to methane [61]. Also, the archaeal community is highly impacted by diet [7]. The archaeal community in the rumen of camels under investigation was dominated by *Methanomethylophilus, Methanobrevibacter*, *Methanosphaera,* and *Methanomicrobium*, this result is consistent with other studies on camel [3],[7]. *Methanobrevibacter* was found dominant in the rumen of a wide range of ruminant animals and correlated with high methane production [54],[62],[63]. Additionally, *Methanomicrobium* correlated positively with high lignocellulose in the animal diet in buffalo [64], and camels [7].

## 5. Conclusions

This study applied Illumina-amplicon sequencing and batch incubation technique to get insight into the composition of bacterial and archaeal communities in the rumen of camels fed low-quality forages and to quantifying the cellulolytic and xylanolytic activities of rumen bacteria. Most of the rumen bacteria in the camel rumen have fibrolytic activities. The production of cellulase and xylanase was impacted by incubation time and cellulose source where the alfalfa hay was associated with high-cellulase production. This study highlights the camel rumen as a promising source for fibrolytic enzymes.

## References

[1] Samsudin AA, Evans PN, Wright AD (2011). Molecular diversity of the foregut bacteria community in the dromedary camel (*Camelus dromedarius*). Environ Microbiol.

[2] Kay RNB, Maloiy GMO (1989). Digestive secretions in camels. Options Méditerranéennes–Série Séminaires-n.°2.

[3] Gharechahi J, Zahiri HS, Noghabi KA (2015). In-depth diversity analysis of the bacterial community resident in the camel rumen. Syst Appl Microbiol.

[4] Lechner-Doll M, Engelhardt WV (1989). Particle size and passage from the forestomach in camels compared to cattle and sheep fed a similar diet. J Anim Physiol Anim Nutr.

[5] Iqbal A, Khan BB (2001). Feeding behaviour of camel. Pak J Agric Sci.

[6] Samsudin AA, Wright ADG, Al Jassim R (2012). Cellulolytic bacteria in the foregut of the dromedary camel (Camelus dromedarius). Appl Environ Microbiol.

[7] Rabee AE, Forster RJ, Elekwachi CO (2020). Comparative analysis of the metabolically active microbial communities in the rumen of dromedary camels under different feeding systems using total rRNA sequencing. Peer J.

[8] Bhatt VD, Dande SS, Patil NV (2013). Molecular analysis of the bacterial microbiome in the forestomach fluid from the dromedary camel (*Camelus dromedarius*). Mol Biol Rep.

[9] Gharechahi J, Salekdeh GH (2018). A metagenomic analysis of the camel rumen's microbiome identifies the major microbes responsible for lignocellulose degradation and fermentation. Biotechnol Biofuels.

[10] Ameri R, Laville E, Potocki-VeÂronèse G (2018). Two new gene clusters involved in the degradation of plant cell wall from the fecal microbiota of Tunisian dromedary. PLoS One.

[11] Jami E, White BA, Mizrahi I (2014). Potential role of the bovine rumen microbiome in modulating milk composition and feed ffficiency. PLoS One.

[12] Moss AR, Jouany JP, Newbold J (2000). Methane production by ruminants: its contribution to global warming. Ann Zootech.

[13] Van Nevel CJ, Demeyer DI (1996). Control of rumen methanogenesis. Environ Monit Assess.

[14] Lee K, Webb RI, Janssen PH (2009). Phylum Verrucomicrobia representatives share a compartmentalized cell plan with members of bacterial phylum Planctomycetes. BMC Microbiol.

[15] Ekinci MS, Özcan N, ÖzkÖse E (2001). A Study on cellulolytic and hemicellulolytic enzymes of anaerobic rumen bacterium *Ruminococcus flavefaciens* Strain 17. Turk J Vet Anim Sci.

[16] Seo JK, Park TS, Kwon IH (2013). Characterization of cellulolytic and xylanolytic enzymes of *Bacillus licheniformis* JK7 isolated from the rumen of a native Korean goat. Asian-Aust J Anim Sci.

[17] Sadhu S, Ghosh PK, Aditya G (2014). Optimization and strain improvement by mutation for enhanced cellulase production by *Bacillus sp*. (MTCC10046) isolated from cow dung. J King Saud UnivSci.

[18] Khatab MSA, Abd El Tawab AM, Fouad MT (2017). Isolation and characterization of anaerobic bacteria from frozen rumen liquid and its potential characterization. Int J Dairy Sci.

[19] Selinger LB, Fosberg CW, Cheng KJ (1996). The rumen: A unique source of enzymes for enhancing livestock production. Anaerobe.

[20] Hess M, Sczyrba A, Egan R (2011). Metagenomic discovery of biomass degrading genes and genomes from cow rumen. Science.

[21] Rabee AE, Al Ahl AAS, Sabra EA (2019a). Assessment of xylanolytic and cellulolytic activities of anaerobic bacterial community in the rumen of camel using different substrates. Menoufia J Animal Poultry Fish Prod.

[22] Molina-Guerrero CE, de la Rosa G, Gonzalez Castañeda J (2018). Optimization of culture conditions for production of cellulase by *Stenotrophomonas maltophilia*. Bio Res.

[23] Sethi S, Datta A, Gupta BL (2013). Optimization of Cellulase Production from Bacteria Isolated from Soil. Inter Scholarly Res Not.

[24] Rabee AE, Forster RJ, Elekwachi CO (2019b). Community structure and fibrolytic activities of anaerobic rumen fungi in dromedary camels. J Basic Microbiol.

[25] Phillips MW, Gordon GLR (1988). Sugar and polysaccharide fermentation by rumen anaerobic fungi from Australia, Britain and New Zealand. BioSystems.

[26] Wang Z, Elekwachi C, Jiao J (2017). Changes in Metabolically Active Bacterial Community during Rumen Development, and Their Alteration by Rhubarb Root Powder Revealed by 16S rRNA Amplicon Sequencing. Front Microbiol.

[27] Liu K, Xu Q, Wang L (2016). Comparative studies of the composition of bacterial microbiota associated with the ruminal content, ruminal epithelium and in the faeces of lactating dairy cows. Microb Biotechnol.

[28] Caporaso JG, Kuczynski J, Stombaugh J (2010). QIIMEE allows analysis of high-throughput community sequencing data. Nat Methods.

[29] Andrews S (2010). Fast QC: a quality control tool for high throughput sequence data.

[30] Bolger AM, Lohse M, Usadel B (2014). Trimmomatic: A flexible trimmer for Illumina Sequence Data. Bioinformatics.

[31] Zhang J, Kobert K, Flouri T (2014). PEAR: a fast and accurate Illumina Paired-End reAd mergeR. Bioinformatics.

[32] Caldwell DR, Bryant MP (1966). Medium without rumen fluid for nonselective enumeration and isolation of rumen bacteria. Appl Microbiol.

[33] McSweeney CS, Denman SE, Mackie RI, Makkar H.P., McSweeney C.S. (2005). Rumen bacteria. Methods in Gut Microbial Ecology for Ruminants.

[34] IBM Corp. Released (2011). IBM SPSS Statistics for Windows, Version 20.0.

[35] Petri RM, Schwaiger T, Penner GB (2013). Characterization of the core rumen microbiome in cattle during transition from forage to concentrate as well as during and after an acidotic challenge. PLoS One.

[36] Pandya PR, Singh KM, Parnerkar S (2010). Bacterial diversity in the rumen of Indian Surti buffalo (*Bubalus bubalis*), assessed by 16S rDNA analysis. J Appl Genet.

[37] Pitta DW, Kumar S, Veiccharelli B (2014). Bacterial diversity associated with feeding dry forage at different dietary concentrations in the rumen contents of Mehshana buffalo (*Bubalus bubalis*) using 16S pyrotags. Anaerobe.

[38] Naas AE, Mackenzie AK, Mravec J (2014). Do rumen Bacteroidetes utilize an alternative mechanism for cellulose degradation?. mBio.

[39] Fouts DE, Szpakowski S, Purushe J (2012). Next generation sequencing to define prokaryotic and fungal diversity in the bovine rumen. PLoS One.

[40] Russell JB, Rychlik JL (2001). Factors that alter rumen microbial ecology. Science.

[41] Nathani NM, Patel AK, Mootapally CS (2015). Effect of roughage on rumen microbiota composition in the efficient feed converter and sturdy Indian Jaffrabadi buffalo (*Bubalus bubalis*). BMC Genomics.

[42] Koike S, Yoshitani S, Kobayashi Y (2003). Phylogenetic analysis of fiber-associated rumen bacterial community and PCR detection of uncultured bacteria. FEMS Microbiol Lett.

[43] Liu K, Xu Q, Wang L (2017). The impact of diet on the composition and relative abundance of rumen microbes in goat. Asian-Australas J Anim Sci.

[44] Gruninger RJ, McAllister TA, Forster RJ (2016). Bacterial and archaeal diversity in the gastrointestinal tract of the orth American beaver (*Castor canadensis*). PLoS One.

[45] Ransom-Jones E, Jones DL, McCarthy AJ (2012). The Fibrobacteres: an important phylum of cellulose-degrading bacteria. Microb Ecol.

[46] Ishaq SL, Wright AG (2012). Insight into the bacterial gut microbiome of the North American moose (*Alces alces*). BMC Microbiol.

[47] Leahy S, Kelly W, Ronimus R (2013). Genome sequencing of rumen bacteria and archaea and its application to methane mitigation strategies. Animal.

[48] Herlemann DPR, Geissinger O, Ikeda-Ohtsubo W (2009). Genomic analysis of “*Elusimicrobium minutum*,” the first cultivated representative of the phylum “Elusimicrobia” (formerly termite group 1). Appl Environ Microbiol.

[49] Ishaq S, Sundset M, Crouse J (2015). High-throughput DNA sequencing of the moose rumen from different geographical locations reveals a core ruminal methanogenic archaeal diversity and a differential ciliate protozoal diversity. Microb Genom.

[50] Zoetendal E, Plugge CM, Akkermans ADL (2003). Victivallisvadensis gen. nov., sp. nov., a sugar-fermenting anaerobe from human faeces. Int J Syst Evol Microbiol.

[51] Jewell KA, McComirck C, Odt CL (2015). Ruminal bacterial community composition in dairy cows is dynamic over the course of two lactations and correlates with feed efficiency. Appl Environ Microbiol.

[52] Noel SJ, Højberg O, Urich T (2016). Draft genome sequence of “Candidatus Methanomethylophilus” sp. 1R26, enriched from bovine rumen, a methanogenic archaeon belonging to the Methanomassiliicoccales order. Genome Announc.

[53] Zorec M, Vodovnik M, MarinŠek-Logar R (2014). Potential of selected rumen bacteria for cellulose and hemicellulose degradation. Food Technol Biotechnol.

[54] Henderson G, Cox F, Ganesh S (2015). Rumen microbial community composition varies with diet and host, but a core microbiome is found across a wide geographical range. Sci Rep.

[55] Carberry CA, Kenny DA, Han S (2012). Effect of phenotypic residual feed intake and dietary forage content on the rumen microbial community of beef cattle. Appl Environ Microbiol.

[56] Shrivastava B, Jain KK, Kalra A (2014). Bioprocessing of wheat straw into nutritionally rich and digested cattle feed. Sci Rep.

[57] Asem D, Leo VV, Passari AK (2017). Evaluation of gastrointestinal bacterial population for the production of holocellulose enzymes for biomass deconstruction. PLoS One.

[58] Salmon DNX, Spier MR, Soccol CR (2014). Analysis of inducers of xylanase and cellulase activities production by *Ganoderma applanatum* LPB MR-56. Fungal Biol.

[59] Williams AG, Withers SE (1982). The production of plant cell wall polysaccharide-degrading enzymes by hemicellulolytic rumen bacterial isolates grown on a range of carbohydrate substrates. J Appl Bacteriol.

[60] Yang W, Meng F, Peng J (2014). Isolation and identification of a cellulolytic bacterium from the Tibetan pig's intestine and investigation of its cellulase production. Electron J Biotechnol.

[61] Hook SE, Wright ADG, McBride BW (2010). Methanogens: methane producers of the rumen and mitigation strategies. Archaea.

[62] St-Pierre B, Wright AG (2012). Molecular analysis of methanogenic archaea in the forestomach of the alpaca (*Vicugna pacos*). BMC Microbiol.

[63] Salgado-Flores A, Bockwoldt M, Hagen L (2016). First insight into the faecal microbiota of the high Arctic muskoxen (*Ovibos moschatus*). Microb Genom.

[64] Franzolin R, Wright AG (2016). Microorganisms in the rumen and reticulum of buffalo (*Bubalus bubalis*) fed two different feeding systems. BMC Research Notes.

