# Supplementary material for: Lignocelluloytic activities and composition of bacterial community in the camel rumen
Source: AIMS Microbiol. 2021 Sep 24;7(3):354–67. doi: 10.3934/microbiol.2021022 (PMC8500796; doi:10.3934/microbiol.2021022)
Supplement: Supplementary file 1 [file microbiol-07-03-022-s001.pdf]

AIMS Microbiology

AIMS Microbiology, 7(3): 354–367.
DOI: 10.3934/microbiol.2021022
Received: 12 July 2021
Accepted: 15 September 2021
Published: 24 September 2021

http://www.aimspress.com/journal/microbiology

Research article

Lignocelluloytic activities and composition of bacterial community in The camel rumen

Alaa Emara Rabee[1,*], Robert Forster[2] and Ebrahim Sabra[3]

[1] Animal and Poultry Nutrition Department, Desert Research Center, Cairo, Egypt
[2] Lethbridge Research and Development Centre, Agriculture and Agrifood Canada, Lethbridge, AB, Canada
[3] University of Sadat City, Sadat City, Egypt

* **Correspondence:** rabee_a_m@yahoo.com; Tel: +201096884139; Fax: +20226357858.

Supplementary

Supplementary Table S1. The chemical composition (%) of diets fed to camels.

Feeds	Chemical analysis (%) on DM basis		
	DM	CP	NDF
Wheat straw	93	2.9	77
Broom corn	90	9	12

Supplementary Table S2. The relative abundances of identified bacterial and archaeal phyla in the rumen of camels.

	Animal A	Animal B	Animal C	Overall mean
Euryarchaeota	0.85	0.46	1.9	1.07
Actinobacteria	0.26	0.36	0.43	0.35
Bacteroidetes	39.99	49.47	39.89	43.1
Candidate division SR1	0.01	0.07	0.22	0.1
Candidate division TM7	0.04	0.02	0.02	0.026

Continued on next page

	Animal A	Animal B	Animal C	Overall mean
Chloroflexi	0.04	0	0.03	0.023
Cyanobacteria	0.09	0.27	0.31	0.22
Elusimicrobia	0.21	5.21	0.14	1.85
Fibrobacteres	22.12	8.44	23.74	18.1
Firmicutes	20.6	26.42	19.56	22.19
Lentisphaerae	0.16	1.5	0.22	0.62
Planctomycetes	0.16	1.5	0.11	0.59
Proteobacteria	1.17	0.75	0.6	0.84
SHA-109	0.22	0.19	0.19	0.2
Spirochaetae	7.79	3.69	7.77	6.41
Synergistetes	0.6	0.72	1.32	0.88

Supplementary Table S3. The relative abundances of bacterial and archaeal genera.

Phylum	Genus	A	B	B	Overall Mean
Euryarchaeota	Methanobrevibacter	0.09	0.11	0.41	0.20
Euryarchaeota	Methanosphaera	0.01	0.08	0.04	0.04
Euryarchaeota	Methanomicrobium	0.03	0.00	0.00	0.01
Euryarchaeota	Candidatus Methanomethylophilus	0.32	0.66	1.45	0.81
Actinobacteria	Corynebacterium	0.03	0.01	0.00	0.02
Actinobacteria	Atopobium	0.12	0.06	0.34	0.17
Bacteroidetes	BS11 gut group;Other	3.81	0.43	1.39	1.88
Bacteroidetes	BS11 gut group;Other	0.46	0.95	0.56	0.66
Bacteroidetes	BS11 gut group;Other	0.08	0.00	0.26	0.11
Bacteroidetes	Prevotellaceae;D_5__Paraprevotella	0.03	0.02	0.03	0.03
Bacteroidetes	Prevotellaceae;D_5__Prevotella	16.90	18.90	15.19	17.00
Bacteroidetes	Prevotellaceae;D_5__uncultured	4.37	13.77	11.50	9.88
Bacteroidetes	Prevotellaceae;D_5__uncultured bacterium	0.01	0.01	0.10	0.04
Bacteroidetes	Prevotellaceae;Other	0.09	0.04	0.04	0.05
Bacteroidetes	RC9 gut group	4.76	1.95	1.15	2.62
Bacteroidetes	S24-7;D_5__uncultured bacterium	0.15	0.34	1.39	0.63
Bacteroidetes	S24-7;D_5__uncultured rumen bacterium	0.38	1.25	6.02	2.55
Bacteroidetes	S24-7;Other	0.01	0.00	0.03	0.01
Cyanobacteria	uncultured bacterium	0.24	0.09	0.17	0.16
Elusimicrobia	Elusimicrobium	5.13	0.21	0.11	1.81
_Fibrobacteres	Fibrobacter	8.42	21.94	23.52	17.96
Firmicutes	Streptococcus	0.01	0.01	0.01	0.01
Firmicutes	Family XIII;D_5__uncultured	0.17	0.09	0.22	0.16
Firmicutes	Acetitomaculum	0.20	0.15	0.46	0.27
Firmicutes	Blautia	0.72	1.01	1.02	0.92
Firmicutes	Butyrivibrio	1.14	2.74	1.62	1.84
Firmicutes	Lachnospiraceae;D_5__Incertae Sedis	1.30	1.12	0.76	1.06
Firmicutes	Lachnospira	0.00	0.14	0.08	0.07
Firmicutes	Roseburia	0.15	0.30	0.32	0.26
Firmicutes	_Lachnospiraceae;D_5__uncultured	1.80	2.45	1.27	1.84

Continued on next page

Phylum	Genus	A	B	B	Overall Mean
Firmicutes	Ruminococcaceae;D_5__Anaerotruncus	0.03	0.02	0.04	0.03
Firmicutes	Ruminococcaceae;D_5__Fastidiosipila	0.01	0.00	0.00	0.00
Firmicutes	Ruminococcaceae;D_5__Incertae Sedis	0.30	0.55	0.17	0.34
Firmicutes	Ruminococcaceae;D_5__Papillibacter	0.32	0.52	0.27	0.37
Firmicutes	Ruminococcaceae;D_5__Ruminococcus	7.65	4.28	2.73	4.89
Firmicutes	Ruminococcaceae;D_5__Saccharofermentans	0.39	0.15	0.26	0.27
Firmicutes	Ruminococcaceae;D_5__uncultured	8.13	1.90	3.11	4.38
Firmicutes	Ruminococcaceae;Other	0.07	0.03	0.05	0.05
Firmicutes	Quinella	0.07	0.01	1.19	0.42
Lentisphaerae	Victivallis	1.16	0.04	0.02	0.41
Planctomycetes	Planctomycetaceae;D_5__CPla-4 termite group	0.07	0.58	0.09	0.25
Proteobacteria	Thalassospira	0.09	0.01	0.01	0.04
Proteobacteria	Desulfovibrio	0.02	0.11	0.10	0.08
Proteobacteria	Campylobacter	0.07	0.04	0.08	0.06
Proteobacteria	Succinimonas	0.01	0.02	0.03	0.02
Proteobacteria	Moraxella	0.02	0.01	0.01	0.01
Proteobacteria	Pseudomonas	0.02	0.04	0.01	0.03
Spirochaetae	Spirochaeta	0.02	0.01	0.00	0.01
Spirochaetae	Treponema	3.67	7.73	7.68	6.36
Spirochaetae	Spirochaetaceae;D_5__uncultured rumen bacterium	0.00	0.04	0.02	0.02
Synergistetes	Pyramidobacter	0.66	0.48	0.95	0.70
Synergistetes	Synergistaceae;D_5__uncultured	0.06	0.12	0.37	0.18
Tenericutes	Anaeroplasma	0.02	0.05	0.04	0.04
Verrucomicrobia	uncultured bacterium	2.54	0.31	0.10	0.98

Supplementary Table S4. Cellulase (endo-cellulase) activity (mU / ml) (mean ± SD) of ruminal bacterial community of the dromedary camel using different cellulosic substrates at different fermentation periods.

	24 h		48 h		7 d	
	Mean	SD	Mean	SD	Mean	SD
Filter paper (FP)	20.6	1.2	96.3	2.6	266.7	23.9
Wheat straw (WS)	82.4	0.6	193.25	14.7	164	19.6
Alfalfa hay (AH)	839.5	25.8	941.8	185	1049.5	22

AIMS Press

© 2021 the Author(s), licensee AIMS Press. This is an open access article distributed under the terms of the Creative Commons Attribution License (http://creativecommons.org/licenses/by/4.0)
